# How to perform Doppler examination of the maternal ophthalmic artery

**DOI:** 10.1002/uog.70346

**Published:** 2026-10-07

**Authors:** A. Youssef, M. Paoli, K. H. Nicolaides

**Affiliations:** ^1^ Obstetrics Unit, IRCCS Azienda Ospedaliero‐Universitaria di Bologna Bologna Italy; ^2^ Harris Birthright Research Centre for Fetal Medicine Kings College Hospital London UK

**Keywords:** Doppler, ophthalmic artery, pre‐eclampsia, screening

How to… Practical advice on imaging‐based techniques and investigations with accompanying videoclips online

## BACKGROUND

The ophthalmic artery is the first branch of the internal carotid artery and provides a practical, non‐invasive window into maternal cerebral hemodynamics. Doppler assessment of this vessel is technically simple, rapid and widely applicable. It offers information that is complementary to uterine artery (UtA) Doppler, as it reflects systemic hemodynamic changes and cerebrovascular adaptation rather than placental perfusion alone^1^, ^2^, ^3^, ^4^.

Early studies have demonstrated that pregnancies complicated by pre‐eclampsia are characterized by reduced impedance and increased flow velocity in the ophthalmic artery, consistent with cerebral vasodilation and impaired autoregulation^5^, ^6^, ^7^, ^8^. The ophthalmic artery waveform has a characteristic morphology with two systolic peaks: the first peak primarily reflects cardiac ejection, while the second peak is influenced by arterial wave reflection and systemic vascular properties. The ratio of the second to the first ophthalmic artery peak systolic velocity (PSV), as described by Nakatsuka *et al*.^9^, has emerged as the most clinically informative Doppler index in the assessment of maternal cerebral hemodynamics in women at risk of pre‐eclampsia^3^, ^9^, ^10^, ^11^, ^12^, ^13^. In pregnancies complicated by pre‐eclampsia, altered maternal systemic hemodynamics and impaired cerebral autoregulation are associated with reduced vascular resistance and increased arterial wave reflection, leading to relative augmentation of the second systolic peak and an increased PSV ratio.

Large, prospective, observational studies have substantially refined both the methodology and clinical interpretation of ophthalmic artery Doppler assessment^10^, ^11^, ^12^, ^13^, ^14^, ^15^, ^16^. In unselected populations assessed at 19–23 weeks' gestation, the ophthalmic artery PSV ratio is increased in pregnancies that subsequently develop pre‐eclampsia, with greater deviation from normal in cases of earlier‐onset disease, and provides complementary information to established biomarkers such as UtA pulsatility index, mean arterial pressure and placental growth factor, improving the prediction of pre‐eclampsia when incorporated into multivariable models^14^, ^15^, ^16^, ^17^. Similar incremental predictive value of the ophthalmic artery PSV ratio has been demonstrated at 35–37 weeks' gestation, including the prediction of imminent pre‐eclampsia^11^, ^12^, ^13^.

First‐trimester data indicate that ophthalmic artery Doppler abnormalities may already be detectable at 11–13 weeks' gestation^18^, ^19^. The PSV ratio has been shown to be increased in first‐trimester pregnancies that later develop pre‐eclampsia, with a stronger association with preterm disease, and it provides a modest but independent improvement in prediction beyond that of maternal factors and established biomarkers^18^, ^19^.

Methodological studies have shown that variability in ophthalmic artery Doppler measurements is greater between the two eyes than between repeated measurements within the same eye. This likely reflects physiological and technical differences between the two sides, and supports the need for bilateral assessment to improve measurement reliability. Consequently, bilateral assessment is recommended. Averaging at least one measurement from each eye minimizes measurement variability, while precision can be further improved by obtaining two measurements per eye when feasible. These findings underline the importance of a standardized vessel identification and acquisition technique to ensure reproducible assessment of ophthalmic artery Doppler indices^12^, ^13^, ^19^. A recent prospective study further explored the relationship of maternal ophthalmic artery Doppler with UtA Doppler, maternal hemodynamic indices and gestational age, and demonstrated no significant association between ophthalmic artery and UtA Doppler indices, suggesting that the two assessments reflect different aspects of maternal cardiovascular adaptation^20^.

In this context, standardized acquisition and interpretation of ophthalmic artery Doppler are essential to obtain reliable and clinically useful measurements. The aim of this How To article is to provide a standardized, step‐by‐step guide for the safe and correct identification and Doppler assessment of the ophthalmic arteries, and the reproducible measurement of ophthalmic artery Doppler indices.

## PRACTICAL POINTS

The technique is summarized in Videoclips S1 and S2.

1. The woman is examined in the supine position after resting for approximately 5 min.
2. After application of ultrasound gel, the transducer is placed transversely and gently over the closed upper eyelid, with care taken to apply only minimal pressure (Figure 1). A high‐frequency linear transducer (approximately 6–15 MHz) is preferred.
3. Color flow imaging is used to identify the ophthalmic artery, which is located superior and medial to the hypoechoic band representing the optic nerve (Figure 2).
4. Pulsed‐wave Doppler is used to record three to five consecutive, similar waveforms under standardized conditions: angle of insonation maintained at < 20°, with angle correction applied when needed; sample gate of approximately 2–4 mm, encompassing the entire longitudinally visualized vessel segment; depth set at 3.0–4.5 cm; high‐pass filter set at 50 Hz; and pulse repetition frequency set at 3–6 kHz.
5. To minimize any potential adverse effects on the eye, Doppler acquisition should be brief, and each eye should be examined for only a few seconds using a dedicated, low‐output orbital preset, with a maximum mechanical index of 0.3. When available, the thermal index should also be kept as low as reasonably achievable, in line with current safety recommendations.
6. The ophthalmic artery waveform is characterized by two systolic peaks (Figure 3). The following indices should be recorded for analysis: first PSV; second PSV; pulsatility index; and PSV ratio. The first PSV and the pulsatility index can be obtained automatically by the ultrasound system, depending on the equipment used, while the second PSV is usually measured manually. The PSV ratio can be calculated subsequently.
7. For reproducible assessment of the ophthalmic artery, measurements should be obtained in both eyes (Figure 3). Acquisition is performed sequentially and, when feasible, measurements are obtained from the right eye followed by the left eye, and then repeated in the right eye followed by the left eye. The recommended approach is to calculate the average of these four measurements (two from each eye). When this is not feasible, the average of one measurement from each eye provides acceptable reliability.

**Figure 1:**
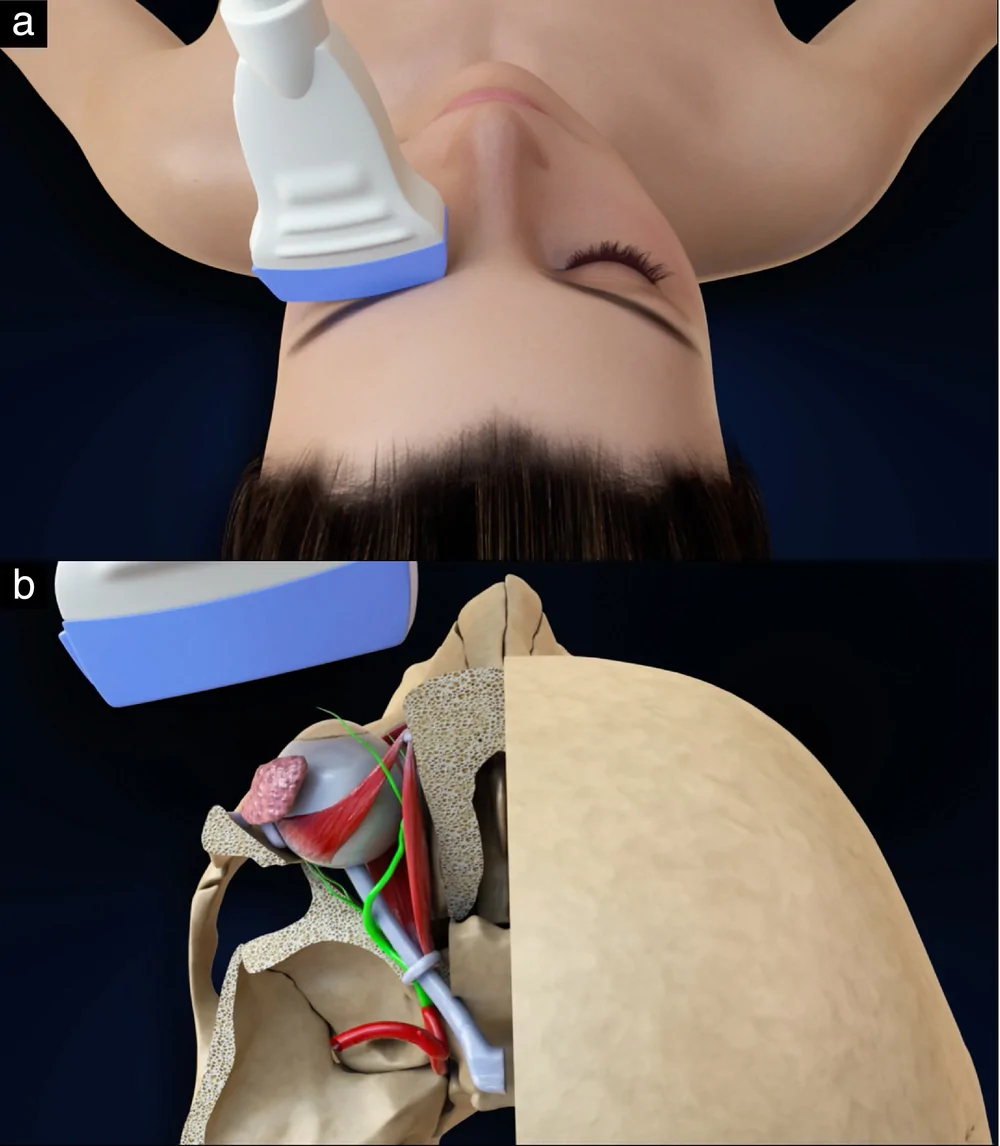
(a) Graphic illustration showing transverse positioning of transducer over closed upper eyelid for ophthalmic artery Doppler assessment, with woman in supine position. (b) Corresponding orbital anatomy, with ophthalmic artery highlighted in green.

**Figure 2:**
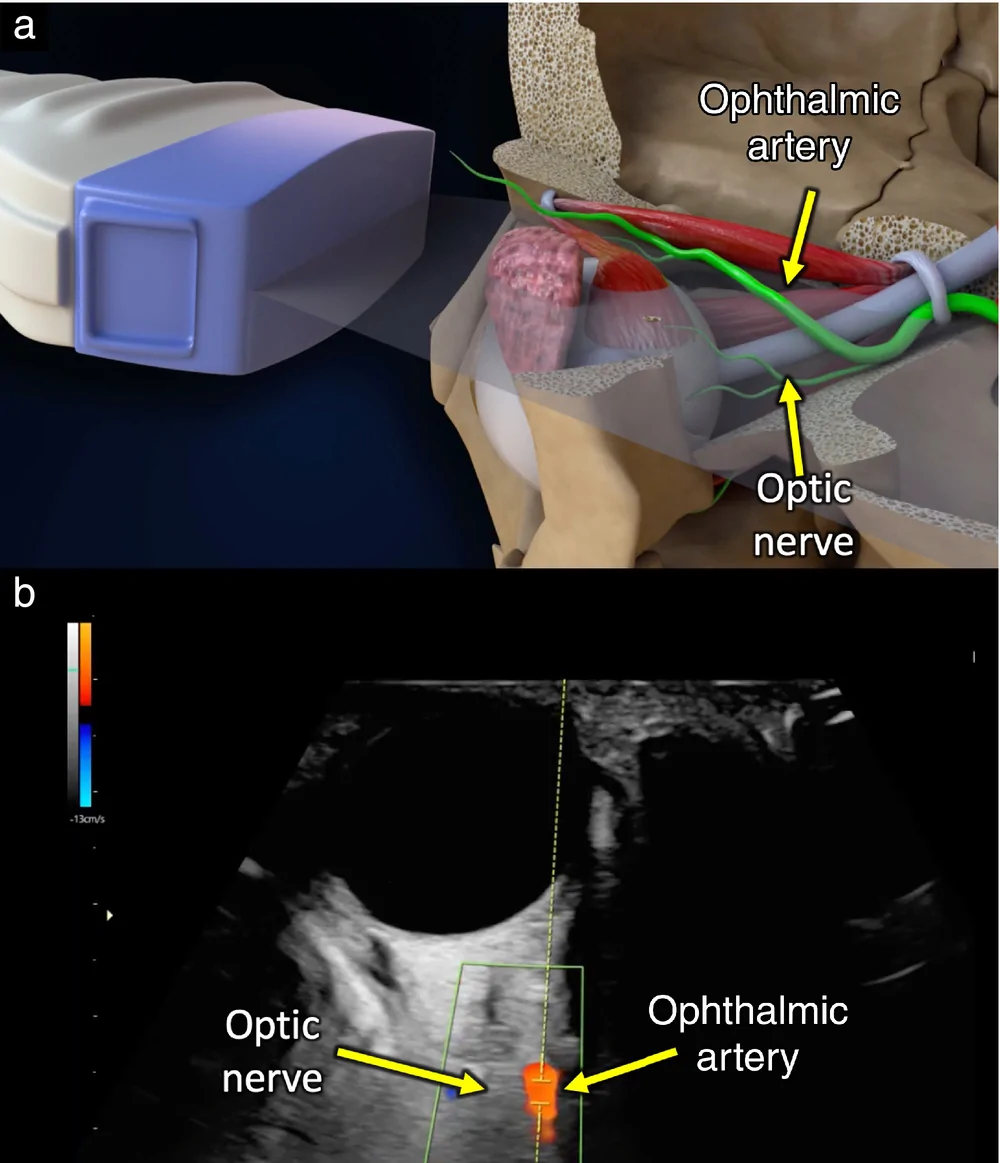
(a) Graphic illustration showing orbital anatomy and transducer orientation, and (b) corresponding ultrasound image with color flow imaging, demonstrating identification of ophthalmic artery medial to the optic nerve and correct placement of the pulsed‐wave Doppler sample volume.

**Figure 3:**
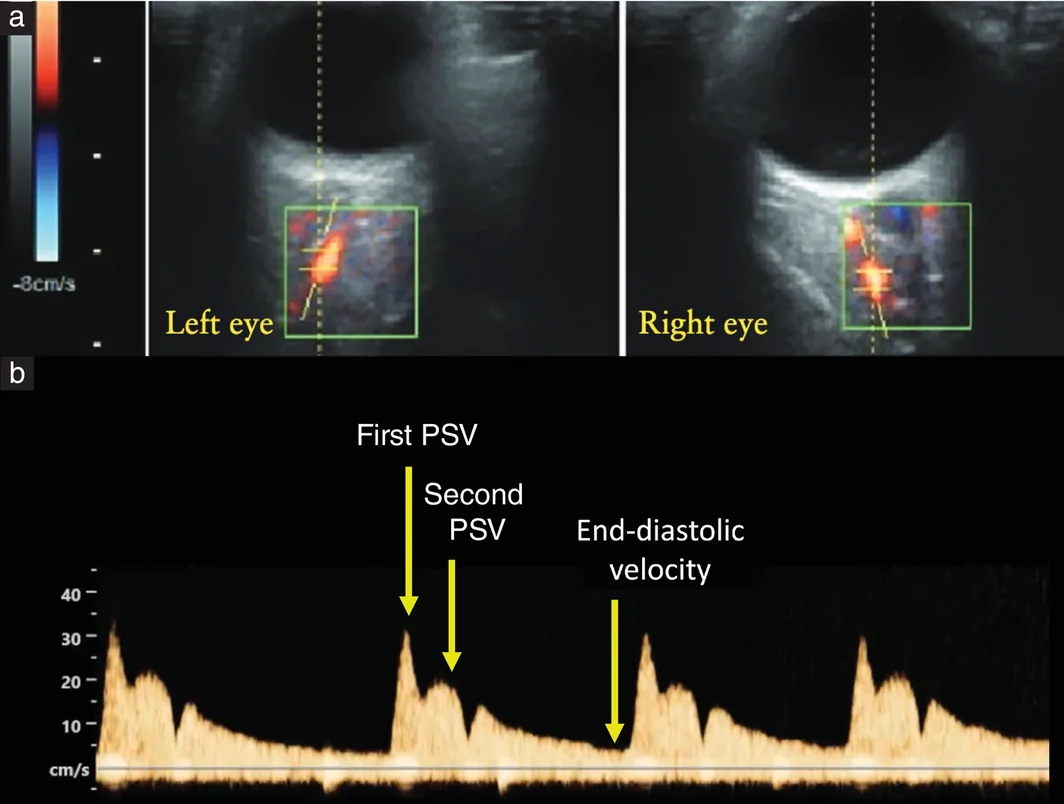
(a) Color flow imaging in left and right eyes, demonstrating bilateral identification of the ophthalmic artery, and (b) corresponding pulsed‐wave Doppler waveform, showing first and second peak systolic velocities (PSVs) and end‐diastolic velocity.

Common pitfalls of ophthalmic artery Doppler measurement include excessive probe pressure applied on the eyelid, which may alter blood flow, as well as incorrect identification of the ophthalmic artery, a suboptimal angle of insonation and motion artifacts. Careful attention to these factors is essential to ensure accurate and reproducible measurements.

### Disclosure

A.Y. has received consultancy fees for lectures delivered for GE Healthcare.

## Supporting information


**Videoclips S1 and S2** Overview of ophthalmic artery Doppler technique, including transducer positioning, vessel identification medial to the optic nerve, and pulsed‐wave Doppler waveform acquisition.

## Data Availability

Data sharing not applicable to this article as no datasets were generated or analysed during the current study.
